# Ligation of MHC Class II Induces PKC-Dependent Clathrin-Mediated Endocytosis of MHC Class II

**DOI:** 10.3390/cells9081810

**Published:** 2020-07-30

**Authors:** Kento Masaki, Yuhji Hiraki, Hiroka Onishi, Yuka Satoh, Paul A. Roche, Satoshi Tanaka, Kazuyuki Furuta

**Affiliations:** 1 Department of Immunobiology, Okayama University Graduate School of Medicine, Dentistry, and Pharmaceutical Sciences, Tsushima naka 1-1-1, Kita-ku, Okayama 700-8530, Japan; pxrp9ufn@s.okayama-u.ac.jp (K.M.); ph422127@s.okayama-u.ac.jp (Y.H.); p75c9g34@s.okayama-u.ac.jp (Y.S.); 2 Department of Immunobiology, Faculty of Pharmacy and Pharmaceutical Sciences, Okayama University, Tsushima naka 1-1-1, Kita-ku, Okayama 700-8530, Japan; pmgj0lp6@s.okayama-u.ac.jp; 3 Experimental Immunology Branch, National Cancer Institute, National Institutes of Health, Bethesda, MD 20892, USA; paul.roche@nih.gov; 4 Department of Pharmacology, Division of Pathological Sciences, Kyoto Pharmaceutical University, Misasagi Nakauchi-cho 5, Yamashina-ku, Kyoto 607-8414, Japan; tanaka-s@mb.kyoto-phu.ac.jp

**Keywords:** MHC-II, dendritic cells, endocytosis, crosslink, PKC

## Abstract

In addition to antigen presentation to CD4^+^ T cells, aggregation of cell surface major histocompatibility complex class II (MHC-II) molecules induces signal transduction in antigen presenting cells that regulate cellular functions. We previously reported that crosslinking of MHC-II induced the endocytosis of MHC-II, which was associated with decreased surface expression levels in murine dendritic cells (DCs) and resulted in impaired activation of CD4^+^ T cells. However, the downstream signal that induces MHC-II endocytosis remains to be elucidated. In this study, we found that the crosslinking of MHC-II induced intracellular Ca^2+^ mobilization, which was necessary for crosslinking-induced MHC-II endocytosis. We also found that these events were suppressed by inhibitors of Syk and phospholipase C (PLC). Treatments with a phorbol ester promoted MHC-II endocytosis, whereas inhibitors of protein kinase C (PKC) suppressed crosslinking-induced endocytosis of MHC-II. These results suggest that PKC could be involved in this process. Furthermore, crosslinking-induced MHC-II endocytosis was suppressed by inhibitors of clathrin-dependent endocytosis. Our results indicate that the crosslinking of MHC-II could stimulate Ca^2+^ mobilization and induce the clathrin-dependent endocytosis of MHC-II in murine DCs.

## 1. Introduction

Dendritic cells (DCs) are potent antigen presenting cells (APCs) widely distributed in peripheral tissues. They capture antigens, such as invaded pathogens, and present the antigen-derived peptides with major histocompatibility antigen class II (MHC-II) molecules on the cell surface. Antigen-specific CD4^+^ T cells recognize the antigen peptide-MHC-II complex (pMHC-II) by T cell receptors (TCRs), which leads to their proliferation and differentiation into effector T cells that initiate an adaptive immune response to the pathogens [1,2,3,4,5].

In addition to their role in antigen presentation, substantial evidence suggests that an aggregation of cell surface MHC-II induces signaling inside the cells by regulating the functioning of APCs, including DCs and B cells [6]. One of the features triggered by MHC-II-induced signaling is the suppression of the potential of antigen presentation in murine bone marrow-derived DCs (BMDCs). MHC-II crosslinking was reported to suppress LPS-induced activation of BMDCs [7]. In addition, we previously reported that crosslinking of MHC-II induces endocytosis and decreases the expression levels of cell surface MHC-II, which results in an attenuation of the potential of antigen presentation [8]. However, the signal transduction and molecular mechanisms that induce endocytosis through MHC-II remain unclear.

Clathrin-dependent endocytosis is one of the pathways through which proteins are transported from the cell surface to intracellular compartments by clathrin-coated vesicles. During this process, the GTPase dynamin is required for the endocytic membrane scission process. Many receptors are internalized through clathrin-dependent and dynamin-dependent endocytosis pathways after ligand binding [9]. On the other hand, some cell surface proteins are internalized by clathrin-independent endocytosis pathways [10,11]. In steady-state DCs, it has been reported that pMHC-II is internalized by clathrin-independent and dynamin-independent pathways [12]. However, it is unclear whether crosslinking-induced endocytosis of MHC-II is attributed to increased steady-state clathrin-independent endocytosis or the induction of an alternative endocytosis route.

In this study, we aimed to investigate the signal transduction pathways involved in crosslinking-induced MHC-II endocytosis in BMDCs.

## 2. Materials and Methods

### 2.1. Materials

The following materials were obtained from the sources indicated: anti-mouse MHC-II antibody (clone 11-5.2) from Biolegend (San Diego, CA, USA); 12-O-tetradecanoyl phorbol 13-acetate (TPA), cytochalasin D, dynasore, chlorpromazine, staurosporine, and piceatannol from Sigma-Aldrich (St. Louis, MO, USA), Fura-2/AM from Dojindo (Kumamoto, Japan), 1,2-bis(o-aminophenoxy)ethane-N,N,N′,N′-tetraacetic acid tetra(acetoxymethyl) ester (BAPTA-AM), U-73122, PD98059, SB203580, and SP600125 from Merck Millipore (Darmstadt, Germany), GF 109203X and R406 from Cayman Chemical (Ann Arbor, MI, USA), recombinant murine granulocyte macrophage colony-stimulating factor (GM-CSF) from Peprotech (Rocky Hill, NJ, USA), anti-FcγRII/RIII (clone 2.4G2) antibody and anti-clathrin heavy chain (CHC) antibody from BD Biosciences (San Jose, CA, USA), Alexa Fluor 488-labeled goat anti-mouse IgG1 antibody and Alexa Fluor 546-labeled goat anti-mouse IgG2b antibody from Thermo Fisher Scientific (Waltham, MA, USA). All other chemicals were commercial products of a reagent grade.

### 2.2. Mice

Specific-pathogen-free, male B10.BR mice (H-2k) were obtained from Japan SLC (Hamamatsu, Japan), and all mice were kept in a specific-pathogen-free animal facility at Okayama University. The Committee on Animal Experiments of Okayama University (approval numbers OKU-2015042 (date of approval: 1 April 2015) and OKU-2018087 (date of approval: 1 April 2018)) approved this study.

### 2.3. Preparation of BMDCs

BMDCs were prepared following the standard protocol [13]. Briefly, bone marrow cells obtained from B10.BR mice were cultured in RPMI-1640 medium containing 10% fetal bovine serum (FBS), 2-mercaptoethanol (50 µM), penicillin (100 units/mL), and streptomycin (100 µg/mL) in the presence of 10 ng/mL GM-CSF for 7 days. More than 90% of the obtained cells were CD11c^+^ and MHC-II^+^ at day 7.

### 2.4. Crosslinking of Cell Surface MHC-II

BMDCs were incubated with an anti-MHC-II antibody (clone 11-5.2, 5 µg/mL) in the presence of an anti-FcγRII/RIII antibody (clone 2.4G2) in FACS staining medium (phosphate-buffered saline (PBS) (−) containing 2% FBS) for 30 min on ice. The cells were washed and surface MHC-II molecules were crosslinked with a biotinylated goat anti-mouse IgG antibody (5 µg/mL) in RPMI-1640 medium containing 10% FBS, 2-mercaptoethanol (50 µM), penicillin (100 units/mL), and streptomycin (100 µg/mL) for 30 min at 37 °C. After washing with FACS staining medium, the cells were incubated with PE-labeled streptavidin for 30 min on ice for detection of the remaining cell surface MHC-II. The fluorescence intensity was measured using a flow cytometer (FACS Calibur, Becton Dickinson, Franklin Lakes, NJ, and Gallios, Beckman Coulter, Brea, CA, USA). The results were presented as a percentage of the fluorescence intensity of the cells placed on ice.

### 2.5. Measurement of Cytosolic Ca^2+^ Concentrations

Cells were loaded with 6 µM Fura-2/AM in modified Tyrode’s buffer (10 mM HEPES-NaOH, pH 7.3, 130 mM NaCl, 5 mM KCl, 1.4 mM CaCl_2_, 1 mM MgCl_2_, 5.6 mM glucose, and 0.1% bovine serum albumin (BSA)) containing 2.5 mM probenecid for 45 min at room temperature. Fluorescence intensities were measured with a fluorescence spectrometer (CAF-110, Jasco, Tokyo, Japan) with an excitation wavelength of 340 or 380 nm and an emission wavelength of 510 nm. Cytosolic Ca^2+^ concentration was expressed as the 340 nm/380 nm ratio.

### 2.6. Immunofluorescence Microscopy

Immunofluorescence microscopy analyses were performed as described previously [14]. BMDCs were seeded on poly-l-lysine coated glass cover slips and allowed to adhere for 30 min at room temperature. The cells were incubated with an anti-MHC-II antibody (clone 11-5.2, 5 µg/mL) for 30 min at 4 °C. The cells were washed and then crosslinked with an Alexa Fluor 546-labeled anti-mouse IgG2b antibody (5 µg/mL) for the periods indicated at 37 °C. The cells were fixed with 4% paraformaldehyde in PBS for 20 min at 4 °C and permeabilized with 0.1% saponin in PBS at room temperature. After the cells were blocked with 2% normal goat serum for 1 h at room temperature, the cells were stained with an anti-CHC antibody for 1 h at 4 °C. The cells were washed and stained with an Alexa Fluor 488-labeled anti-mouse IgG1 antibody as the secondary antibody. The cells were visualized using confocal laser scanning microscopy (OLYMPUS FV300 and FV1200, Tokyo, Japan). For quantitative fluorescence analysis, Manders’ overlay coefficients were calculated using ImageJ software with the Coloc2 plugin, using at least 10 cells in each analysis [15].

### 2.7. Statistical Analysis

Data are presented as the mean ± SEM. Statistical significance for comparisons between the two groups was determined using the Student’s t-test. Statistical significance for comparisons among multiple groups was determined using one-way ANOVA. Additional comparisons were made using Dunnett’s Multiple Comparison test to compare with the control groups, or the Tukey-Kramer Multiple Comparison test for all pairs of column comparisons.

## 3. Results

### 3.1. Crosslinking-Induced Intracellular Ca^2+^ Mobilization is Required for Endocytosis of MHC-II

**Figure 1:**
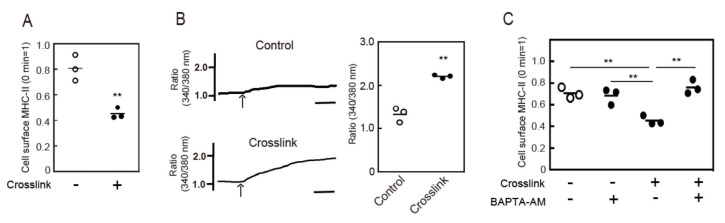
Intracellular Ca^2+^ mobilization is required for crosslinking-induced endocytosis of major histocompatibility complex class II (MHC-II). (**A**) Bone marrow-derived dendritic cells (BMDCs) were incubated with an anti-MHC-II antibody for 30 min at 4 °C. For crosslinking, the cell surface MHC-II was incubated with a biotin-labeled anti-mouse IgG antibody for 30 min at 37 °C. The remaining cell surface MHC-II was detected using PE-labeled streptavidin. (**B**) BMDCs were crosslinked with an anti-MHC-II antibody and an anti-mouse IgG antibody. The cytosolic Ca^2+^ mobilization after stimulation was detected using Fura-2/AM. Bars = 1 min. Relative changes in the cytosolic Ca^2+^ concentrations are expressed as the ratio of fluorescence intensity at 340/380 nm. (**C**) BMDCs were pretreated with vehicle (0.1% dimethyl sulfoxide (DMSO)) or BAPTA-AM (50 μM) for 30 min at 37 °C. The cell surface MHC-II was crosslinked with an anti-MHC-II antibody for 30 min. The remaining cell surface MHC-II was detected by flow cytometry. Values of ** *p* < 0.01 are regarded as significant.

As previously reported [8], MHC-II was spontaneously internalized, and crosslinking of MHC-II enhanced a down-regulation of cell surface MHC-II expression in BMDCs (Figure 1A). In this case, we investigated the signaling mechanisms involved in the endocytosis of MHC-II. Since MHC-II crosslinking was reported to induce intracellular Ca^2+^ mobilization in B cells [16,17,18], we measured the cytosolic Ca^2+^ levels after MHC-II crosslinking in BMDCs and found that MHC-II crosslinking induced Ca^2+^ mobilization (Figure 1B). We then examined the effects of cytosolic Ca^2+^ depletion in spontaneous and crosslinking-induced endocytosis of MHC-II using a Ca^2+^ chelator, BAPTA-AM. While BAPTA-AM treatment did not affect the spontaneous (crosslink (−)) endocytosis of MHC-II, it inhibited the crosslinking-induced endocytosis of MHC-II (Figure 1C). We confirmed that BAPTA-AM treatment did not affect the distribution of MHC-II expression in BMDCs (Figure S1). These results indicate that intracellular Ca^2+^ mobilization is required for crosslinking-induced endocytosis of MHC-II.

### 3.2. Intracellular Ca^2+^ Mobilization Induces Endocytosis of MHC-II

**Figure 2:**
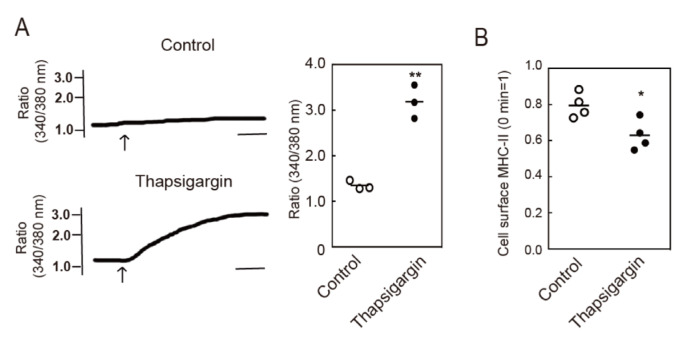
Intracellular Ca^2+^ mobilization induces endocytosis of MHC-II. (**A**) BMDCs were stimulated with or without thapsigargin and cytosolic Ca^2+^ mobilization was detected using Fura-2/AM. Bars = 1 min. Relative changes in the cytosolic Ca^2+^ concentrations are expressed as the ratio of fluorescence intensity at 340/380 nm. (**B**) BMDCs were incubated with an anti-MHC-II antibody for 30 min at 4 °C. The cells were washed and stimulated with thapsigargin for 30 min at 37 °C. The remaining cell surface anti-MHC-II antibody was detected using a PE-labeled anti-mouse IgG antibody. Values of * *p* < 0.05, ** *p* < 0.01 are regarded as significant.

Next, we investigated whether intracellular Ca^2+^ mobilization accelerates the endocytosis of MHC-II by examining the effects of a reagent that induces intracellular Ca^2+^ mobilization. An endoplasmic reticulum calcium ATPase inhibitor, thapsigargin, is known to induce Ca^2+^ mobilization in many cell types and we confirmed that thapsigargin induced intracellular Ca^2+^ mobilization in BMDCs (Figure 2A). We then investigated the effects of thapsigargin on MHC-II endocytosis and found that thapsigargin treatment induced significant levels of endocytosis of MHC-II (Figure 2B). These results suggest that intracellular Ca^2+^ mobilization can induce MHC-II endocytosis.

### 3.3. Crosslinking-Induced Endocytosis of MHC-II is Syk and Phospholipase C (PLC)-Dependent

**Figure 3:**
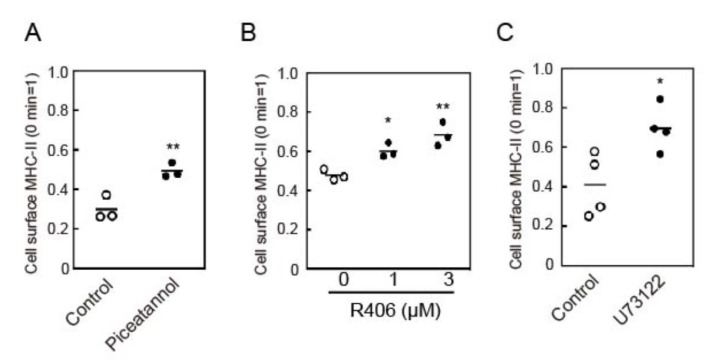
Effects of Syk and phospholipase C (PLC) inhibitors on crosslinking-induced MHC-II endocytosis. BMDCs were pre-incubated with (**A**) piceatannol (50 μM), (**B**) R406 (indicated concentrations), or (**C**) U73122 (10 μM) for 30 min at 37 °C. The cell surface MHC-II was crosslinked with an anti-MHC-II antibody for 30 min. The remaining cell surface MHC-II was detected by flow cytometry. Values of * *p* < 0.05, ** *p* < 0.01 are regarded as significant.

**Figure 4:**
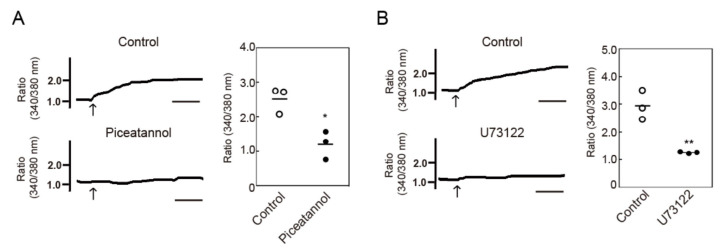
Effects of Syk and PLC inhibitors on MHC-II crosslinking-induced intracellular Ca^2+^ mobilization. BMDCs were pre-incubated with (**A**) piceatannol (50 μM) or (**B**) U73122 (10 μM) for 30 min at 37 °C. The cytosolic Ca^2+^ mobilization after MHC-II crosslinking was measured using Fura-2/AM. Relative changes in the cytosolic Ca^2+^ concentrations are expressed as the ratio of fluorescence intensity at 340/380 nm. Bars = 1 min. Values of * *p* < 0.05, ** *p* < 0.01 are regarded as significant.

Activation of an immunoreceptor tyrosine-based activation motif (ITAM) containing C-type lectin receptor, Dectin-1, was found to induce intracellular Ca^2+^ mobilization, which was mediated by Syk tyrosine kinase-mediated PLCγ activation, in BMDCs [19]. It was also reported that MHC-II interacted with the ITAM-containing adaptor protein, FcγRγ, in BMDCs, and that MHC-II crosslinking induces tyrosine phosphorylation of cellular proteins [7]. Therefore, we examined the contribution of Syk and phospholipase C (PLC) using inhibitors against these molecules. Crosslinking-induced endocytosis of MHC-II was suppressed by Syk inhibitors, (piceatannol and R406), and a PLC inhibitor (U73122) (Figure 3). We then examined the effect of piceatannol and U73122 on intracellular Ca^2+^ mobilization, and found that both inhibitors suppressed Ca^2+^ mobilization induced by MHC-II crosslinking (Figure 4A,B). These results suggest that Syk and PLC are potentially involved in intracellular Ca^2+^ mobilization and MHC-II endocytosis induced by crosslinking of MHC-II.

### 3.4. Protein Kinase C (PKC) Activation is Involved in MHC-II Endocytosis

**Figure 5:**
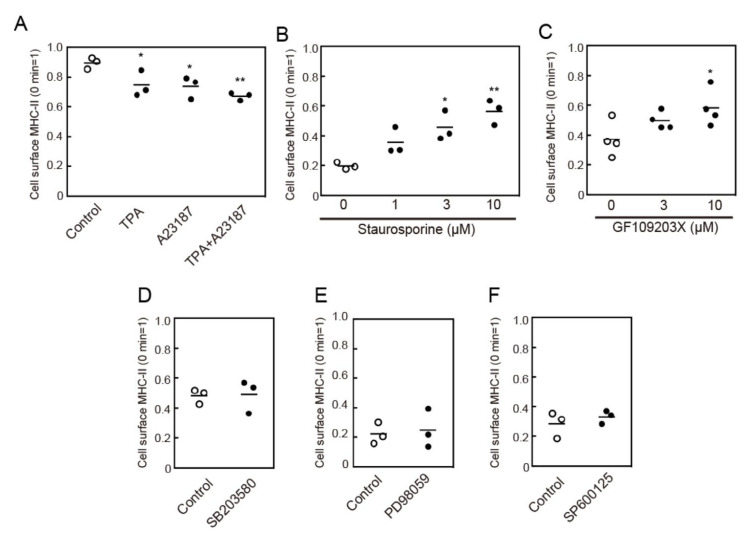
Protein kinase C (PKC) activation induces MHC-II endocytosis. (**A**) BMDCs were incubated with an anti-MHC-II antibody for 30 min at 4 °C. The cells were washed and stimulated with 12-O-tetradecanoyl phorbol 13-acetate (TPA) (100 nM) and A23187 (1 μM) for 30 min at 37 °C. The remaining cell surface anti-MHC-II antibody was detected using a PE-labeled anti-mouse IgG antibody. (**B**,**C**) BMDCs were pre-incubated with staurosporine for 15 min or GF109203X for 30 min at the indicated concentrations of 37 °C. The cell surface MHC-II was crosslinked using an anti-MHC-II antibody. (**D**–**F**) BMDCs were pre-incubated with SB203580 (10 µM) for 30 min, PD98059 (50 µM) for 15 min, or SP600125 (20 µM) for 15 min, at 37 °C. The cell surface MHC-II was crosslinked using an anti-MHC-II antibody for 30 min. The remaining cell surface MHC-II was detected by flow cytometry. Values of * *p* < 0.05, ** *p* < 0.01 are regarded as significant.

One of the signaling molecules activated by intracellular Ca^2+^ mobilization is PKC. We, therefore, examined the effects of Ca^2+^ ionophore, A23,187, and a phorbol ester, TPA, which are known activators of conventional PKC isoforms, on MHC-II endocytosis. Both TPA and A23187 induced MHC-II endocytosis (Figure 5A). Furthermore, pretreatment with broad-spectrum PKC inhibitors, staurosporine, or GF109203X suppressed crosslinking-induced MHC-II endocytosis (Figure 5B,C). These results suggest that Ca^2+^-dependent PKC activation is involved in MHC-II endocytosis.

Activation of mitogen-activated protein (MAP) kinases is required for the endocytosis of several cell surface receptors such as epidermal growth factor receptor (EGFR) [20]. Furthermore, MHC-II-induced signaling has been reported to activate ERK, which is one of the MAP kinases, in BMDCs [7]. Therefore, we investigated the effect of MAP kinase inhibitors on MHC-II endocytosis. Pretreatment with PD98059 (a MEK inhibitor), SB203580 (a p38 MAPK inhibitor), or SP600125 (a JNK inhibitor) did not affect crosslinking-induced MHC-II endocytosis (Figure 5D–F), which suggests that MAP kinases are not involved in this process.

### 3.5. Crosslinking-Induced Endocytosis of MHC-II is a Clathrin-Dependent Response

**Figure 6:**
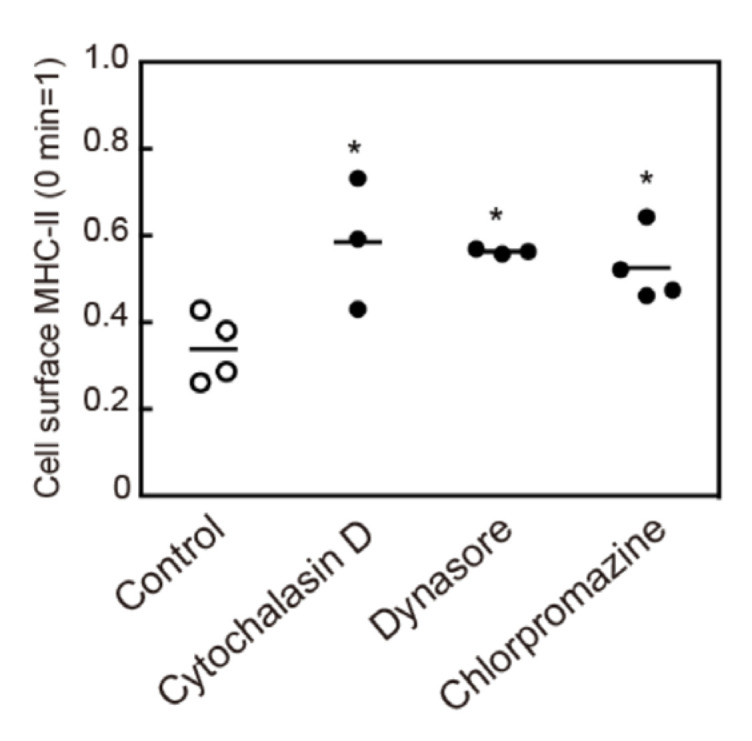
Effect of endocytosis inhibitors on crosslinking-induced MHC-II endocytosis. BMDCs were pre-incubated with cytochalasin D (3 µM), dynasore (100 µM), or chlorpromazine (30 µM), for 30 min at 37 °C. The cell surface MHC-II was crosslinked with an anti-MHC-II antibody for 30 min. The remaining cell surface MHC-II was detected by flow cytometry. Values of * *p* < 0.05 are regarded as significant.

Next, we investigated the endocytosis pathway of MHC-II upon its crosslinking. Cytochalasin D, which inhibits several endocytosis pathways by inhibiting actin polymerization, suppressed crosslinking-induced MHC-II endocytosis (Figure 6). In addition, a dynamin inhibitor, dynasore, and a clathrin-mediated endocytosis inhibitor, chlorpromazine, suppressed crosslinking-induced MHC-II endocytosis (Figure 6). This result suggests that crosslinking-induced MHC-II endocytosis is dependent on both dynamin and clathrin.

**Figure 7:**
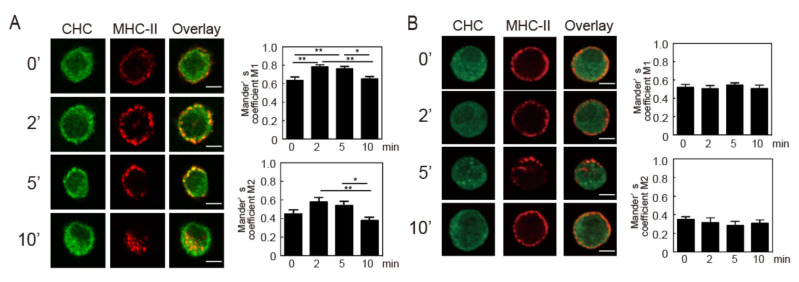
Crosslinking-induced endocytosis of MHC-II is clathrin-dependent. (**A**) BMDCs were incubated with an anti-MHC-II antibody for 30 min at 4 °C. (**B**) BMDCs pre-incubated with chlorpromazine (30 µM) for 30 min at 37 °C were incubated with an anti-MHC-II antibody for 30 min at 4 °C. The cells were washed and crosslinked with an Alexa 546-labeled anti-mouse IgG for the indicated time periods at 37 °C. The cells were fixed, permeabilized, and stained with an anti-clathrin heavy chain (CHC) antibody and an Alexa Fluor 488-labeled secondary antibody. bars = 2µm. Co-localization of MHC-II and CHC was quantified by calculating Manders’ coefficients M1 (green pixels (CHC) overlapping red pixels (MHC-II)) and M2 (red pixels overlapping green pixels) using ImageJ software. Values are presented as the mean ± SEM (n = 10), * *p* < 0.05, ** *p* < 0.01.

**Figure 8:**
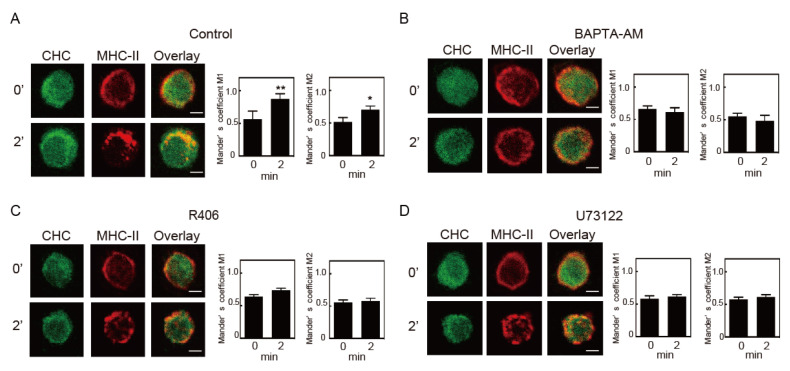
Effect of inhibitors on crosslinking-induced co-localization of MHC-II and clathrin. BMDCs pre-incubated with (**A**) control (0.1% DMSO), (**B**) BAPTA-AM (50 μM), (**C**) R406 (3 μM), or (**D**) U73122 (10 μM) for 30 min at 37 °C. The cell surface MHC-II was crosslinked with anti-MHC-II antibody. The co-localization of MHC-II and CHC was analyzed as described in Figure 7. Values are presented as the mean ± SEM (n = 10), * *p* < 0.05, ** *p* < 0.01.

Clathrin-dependent endocytosis requires a transient interaction between clathrin and the target protein. The degree of co-localization between internalized MHC-II and the clathrin heavy chain (CHC) was quantified by calculating Manders’ overlap coefficient. Increases in co-localization of MHC-II with CHC just beneath the cell surface were observed 2 and 5 min after crosslinking. The majority of the MHC-II signal was found inside the cells 10 min after crosslinking and did not overlap with those of CHC (Figure 7A). In agreement with a previous study [21,22], chlorpromazine treatment prevented redistribution of CHC (Figure 7B). In the cells treated with chlorpromazine, most of the MHC-II remained at the cell surface for 2, 5, and 10 min after crosslinking and co-localization of MHC-II with CHC was hardly observed. These results indicate that crosslinking-induced MHC-II endocytosis is clathrin-dependent. We then investigated the effects of inhibitors against crosslinking-induced signaling on co-localization of MHC-II and clathrin after crosslinking. Similar to the result presented in Figure 7, an increase in co-localization of MHC-II and clathrin was observed after crosslinking for 2 min in control-pretreated cells (Figure 8A). When the BMDCs were pretreated with BAPTA-AM, R406, and U73122, the co-localizations of MHC-II and clathrin was not enhanced (Figure 8B–D). These results suggested that MHC-II crosslinking-induced signaling induces the co-localization of MHC-II and clathrin.

## 4. Discussion

**Figure 9:**
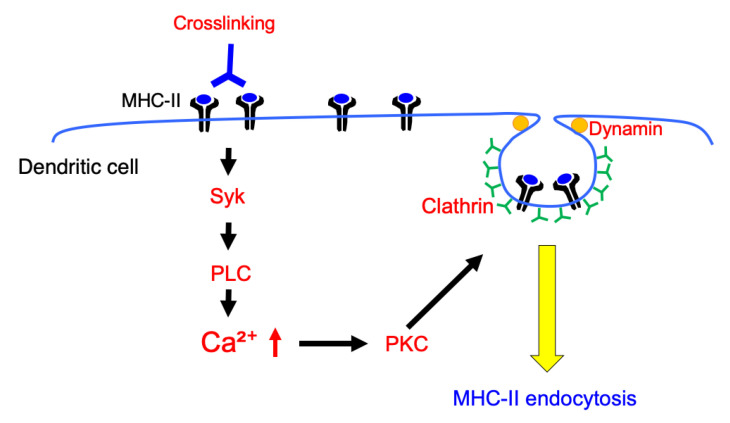
Schematic overview of MHC-II crosslinking induced signaling, which induces MHC-II endocytosis in BMDCs. MHC-II crosslinking induces Syk and PLC-mediated cytosolic Ca^2+^ mobilization. Activation of PKC, which is one of the molecules activated by cytosolic Ca^2+^ mobilization, induces endocytosis of MHC-II. These signal transductions induce colocalization of MHC-II with clathrin, which results in an induction of clathrin-dependent and dynamin-dependent MHC-II endocytosis.

In this study, we investigated the MHC-II crosslinking-induced signaling pathway and molecular mechanisms involved in the endocytosis of MHC-II in murine BMDCs (Figure 9). There are various reports on the function of MHC-II-induced signals [6]. Liang et al. demonstrated that MHC-II crosslinking suppresses Toll-like receptor (TLR) signaling-induced upregulation of CD86 and CD40 in murine BMDCs [7]. They investigated the mechanism responsible for the suppression and found that MHC-II crosslinking induces activation of ERK via the ITAM-containing adapter protein, FcγRγ, which activates a tyrosine phosphatase SHP-1. They also showed that an inhibitor of Syk did not affect the MHC-II crosslinking-induced suppression of TLR signaling. In our study, we investigated the MHC-II crosslinking-induced signaling that induces MHC-II endocytosis, and found that Syk-and PLC-mediated Ca^2+^ mobilization is involved in MHC-II endocytosis. Crosslinking-induced MHC-II endocytosis was not affected by ERK inhibition. These results suggest that MHC-II crosslinking activates multiple signaling pathways and that these signals act in different ways, which might impair the ability of antigen-presentation in BMDCs. Since FcγRγ is known to be involved in the activation of Syk in several cells [23], a similar signaling pathway may be utilized in BMDCs upon MHC-II crosslinking. Alternatively, since MHC-II is reported to interact with other membrane proteins in B cells, such as CD79a and CD79b [17], SCIMP [24], and MPYS/STING [25]. MHC-II might interact with adapter proteins other than FcγRγ to induce Syk/PLC signaling in BMDCs. Further analysis is required to clarify the signal transduction form MHC-II crosslinking to cytosolic Ca^2+^ mobilization.

In this study, we found that MHC-II crosslinking induced Ca^2+^ mobilization and that inhibition of Ca^2+^ mobilization by inhibitors suppressed MHC-II endocytosis. These results suggested that Ca^2+^ mobilization is required for crosslinking-induced MHC-II endocytosis. Although thapsigargin promoted Ca^2+^ mobilization and MHC-II endocytosis, the effect on MHC-II endocytosis was weaker than that on MHC-II crosslinking. Since crosslinking is known to promote the phosphorylation of various proteins in BMDCs [7], MHC-II crosslinking may induce signaling pathways other than Ca^2+^ mobilization that result in enhanced MHC-II endocytosis.

Crosslinking-induced MHC-II endocytosis was inhibited by PKC inhibitors. In addition, endocytosis of MHC-II was promoted by some PKC activators without MHC-II crosslinking. These results suggest that PKC may play an important role in the process. Crosslinking of MHC-II was reported to induce activation of PKCβ in human B cells [26,27], and of PKCδ in human monocyte-derived dendritic cells [28,29]. These PKC isoforms may also be activated in BMDCs by MHC-II crosslinking and regulate the induction of MHC-II endocytosis. Several PKC isoforms are reported to be involved in ligand-induced endocytosis of some membrane proteins. Activation of PKCβII is required for ligand-induced endocytosis of dopamine receptor D3 [30], and activation of PKCα is necessary for the down-regulation of TCRs in antigen-activated T cells. These PKC isoforms may be activated by MHC-II crosslinking and may be involved in endocytosis of MHC-II in BMDCs. Further analysis is required to determine which PKC isoforms could be activated upon crosslinking.

Induction of clathrin-dependent endocytosis is regulated by PKC for several proteins. Endocytosis of TCRs induced by ligand binding is clathrin-dependent [31,32]. In this case, PKC-mediated phosphorylation of Ser 163 of β-arrestin-1 regulates the TCR endocytosis [33,34]. Activation of PKC was reported to induce clathrin-dependent endocytosis of the glutamate transporter, GLT-1, and the human organic cation transporter (hOAT). In this case, a ubiquitin E3 ligase, Nedd 4-2, is activated by PKCs. The activated Nedd 4-2 ubiquitinates GLT-1 and hOAT, which results in their endocytosis [35,36]. Although MHC-II is known to be ubiquitinated at Lys 225, a K225R point-mutation did not affect crosslinking-induced MHC-II endocytosis [8], which suggests that direct ubiquitination of MHC-II is not involved in crosslinking-induced MHC-II endocytosis.

We examined the molecular mechanisms of crosslinking-induced MHC-II endocytosis using several endocytosis inhibitors. Crosslinking-induced endocytosis was suppressed by chlorpromazine and dynasore, which indicates that crosslinking-induced MHC-II endocytosis is clathrin and dynamin-dependent. The signaling inhibitors, BAPTA-AM, R406, and U73122, also suppressed co-localization of MHC-II and clathrin. Crosslinking-induced signals likely promote the transition of an MHC-II to the clathrin-dependent endocytosis pathway.

In steady-state DCs, MHC-II is reported to internalize via clathrin-independent and dynamin-independent pathways [12]. We found that BAPTA-AM treatment did not affect the steady-state endocytosis of MHC-II. These results suggest that crosslinking does not simply accelerate the existing endocytosis pathway but induces an alternative endocytosis pathway. We previously found that crosslinking-induced MHC-II endocytosis was inhibited by methyl-β-cyclodextrin (MCD) treatment, which raises the possibility that lipid rafts could be involved in the process [8]. Although it is well known that MCD blocks clathrin-independent endocytosis, disruption of lipid rafts is also reported to inhibit the clathrin-dependent endocytosis of some proteins. For example, endocytosis of B cell receptors (BCRs) after crosslinking is suppressed by disruption of lipid rafts, even though endocytosis of BCRs is clathrin-dependent in B cells [37,38]. MCD treatment may disrupt the association of signaling molecules in the lipid raft, which results in inhibition of intracellular signal transduction triggered by MHC-II ligation.

In summary, we investigated the molecular mechanisms that could potentially regulate crosslinking-induced MHC-II endocytosis. Our results suggested that MHC-II crosslinking-induced intracellular Ca^2+^ mobilization is required to induce MHC-II endocytosis, and that crosslinking-induced MHC-II endocytosis is clathrin and dynamin-dependent. Since MHC-II endocytosis was suppressed by PKC inhibitors and promoted by PKC activators, intracellular Ca^2+^ mobilization by MHC-II crosslinking may induce activation of PKCs. This activation results in induction of MHC-II endocytosis. The present study suggested that intracellular Ca^2+^ mobilization could be one of the factors determining the cell surface expression levels of MHC-II in DCs.

## Supplementary Materials

The following are available online at https://www.mdpi.com/2073-4409/9/8/1810/s1. Figure S1: Effect of crosslinking and BAPTA-AM on endocytosis of MHC-II.
