# Supplementary material for: Ligation of MHC Class II Induces PKC-Dependent Clathrin-Mediated Endocytosis of MHC Class II
Source: Cells. 2020 Jul 30;9(8):1810. doi: 10.3390/cells9081810 (PMC7465434; doi:10.3390/cells9081810)
Supplement: Supplementary file 1 [file cells-09-01810-s001.pdf]

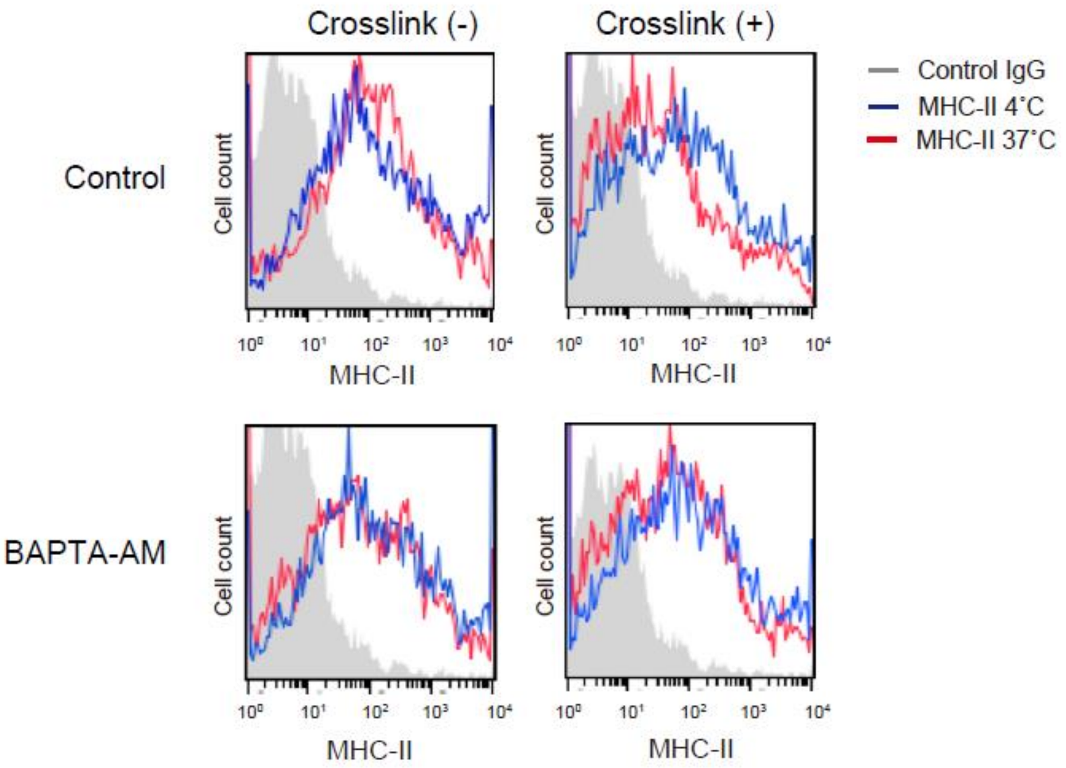

Figure S1 Effect of crosslinking and BAPTA-AM on endocytosis of MHC-II. BMDCs were pretreated with or without BAPTA-AM (50 μM) for 30 min at 37 °C. The cells were then incubated with (crosslink (+)) or without (Crosslink (-)) a primary anti-MHC-II antibody for 30 min at 4 °C. The cell surface MHC-II antibody was crosslinked with a secondary antibody for 30 min at 37 °C (red line) or 4 °C (blue line). The remaining cell surface MHC-II antibody was detected by flow cytometry. Gray filled histogram indicates control IgG staining.
